# Supplementary material for: Exploring the delivery of empathic care in task-shared settings: A psychometric study in rural Pakistan
Source: Glob Ment Health (Camb). 2025 Jan 13;12:e15. doi: 10.1017/gmh.2025.4 (PMC11867816; doi:10.1017/gmh.2025.4)
Supplement: Liaquat et al. supplementary material [file S2054425125000044sup001.docx]

**Supplementary table 1: Empathy Scale for Lay-Therapists (ESLT)**

| جیسا کہ ہیلتھ ورکر یا رضاکار کی طرف سے صحت مند سوچ پروگرام کے سیشنز دیے گئے ہیں۔ یہ سوالنامہ یہ جاننے کےلئے بنایا گیا ہے کہ آپکی ہیلتھ ورکر یا رضاکار اُن سیشنز کو کِس حد تک بہتر اور ہمدردانہ طریقے سے آپ تک پہنچانے میں کامیاب رہی۔اُن سیشنز / ملاقات کو ذہن میں رکھتے ہوئے مندرجہ ذیل سوالات کے ۱ سے ۴ تک کے پیمانے پہ جوابات دیں۔ | | |
| --- | --- | --- |
| **Sr. No,** | **Item** | **Rating** |
|  | Did the health worker or peer talk to you in a way that was easy to understand?  کیا ہیلتھ ورکر یا رضاکار نے آپ سے آسان اور سادہ زبان میں بات کی؟ | 0. Strongly disagree مکمل طور پر غیر متفق  1. Disagree غیر متفق  2. Agree متفق  3. Strongly agree مکمل طور پر متفق |
|  | Did the health worker or peer show respect to you?  کیا ہیلتھ ورکر یا رضاکار آپ سے باعزت طریقے سے پیش ائی؟ | 0. Strongly disagree مکمل طور پر غیر متفق  1. Disagree غیر متفق  2. Agree متفق  3. Strongly agree مکمل طور پر متفق |
|  | Did you feel that your health worker or peer was compassionate towards you?  کیا آپکوایسا محسوس ہوا کہ آپکی ہیلتھ ورکر یا رضاکار آپکی ہمدرد ہیں؟ | 0. Strongly disagree مکمل طور پر غیر متفق  1. Disagree غیر متفق  2. Agree متفق  3. Strongly agree مکمل طور پر متفق |
|  | Did you feel that you can trust your health worker or peer?  کیا آپکوایسا لگا کہ آپ اپنی ہیلتھ ورکر یا رضاکار پہ اعتبار کر سکتی ہیں؟ | 0. Strongly disagree مکمل طور پر غیر متفق  1. Disagree غیر متفق  2. Agree متفق  3. Strongly agree مکمل طور پر متفق |
|  | Did you feel that your health worker or peer listened to you with full attention (e.g. through her eye contact, body language, and posture)?  کیا آپ نے محسوس کیا کہ ہیلتھ ورکر یا رضاکار آپکی باتوں کو پوری توجہ سے سنتی تھی (جیسا کہ: اُس کے دیکھنے کا انداز، چہرے کے تاثرات اور جسمانی سکنات اور انداز وغیرہ )؟ | 0. Strongly disagree مکمل طور پر غیر متفق  1. Disagree غیر متفق  2. Agree متفق  3. Strongly agree مکمل طور پر متفق |
|  | Did the health worker or peer use open ended questions?  کیا ہیلتھ ورکر یا رضاکار نےآپ سے ایسے سوال کیے جن سے آپ کو مزید تفصیل میں بات کرنے کا موقع ملا ؟ | 0. Strongly disagree مکمل طور پر غیر متفق  1. Disagree غیر متفق  2. Agree متفق  3. Strongly agree مکمل طور پر متفق |
|  | Did the health worker or peer acknowledge your feelings?  کیا ہیلتھ ورکر یا رضا کار نے آپکو بتایا کہ وہ آپ کے احساسات اور جذبات کو سمجھ رہی ہے؟ | 0. Strongly disagree مکمل طور پر غیر متفق  1. Disagree غیر متفق  2. Agree متفق  3. Strongly agree مکمل طور پر متفق |
|  | Did the health worker or peer summarize what you had said to help you know that she has understood you?  کیا ہیلتھ ورکر یا رضاکار نےآپ کی باتوں کو مختصراً دہرایا, جِس سے آپ کو یہ جاننے میں مدد ملی کہ وہ آپکی بات کو پوری طرح سے سمجھ گئی ہے؟ | 0. Strongly disagree مکمل طور پر غیر متفق  1. Disagree غیر متفق  2. Agree متفق  3. Strongly agree مکمل طور پر متفق |
|  | Did your health worker or peer try to understand your health problems and their impact on you?  کیا ہیلتھ ورکر یا رضاکار نے آپ کی صحت سے متعلق مشکلات اور اُن کے اثرات کو جاننے کی کوشش کی ؟ | 0. Strongly disagree مکمل طور پر غیر متفق  1. Disagree غیر متفق  2. Agree متفق  3. Strongly agree مکمل طور پر متفق |
|  | Did the health worker or peer tell you that in difficult situations such emotions can be experienced?  کیا ہیلتھ ورکر یا رضا کار نے آپ کو بتایا کہ مشکل حالات میں ایسے جذبات محسوس کیے جاسکتے ہیں ؟ | 0. Strongly disagree مکمل طور پر غیر متفق  1. Disagree غیر متفق  2. Agree متفق  3. Strongly agree مکمل طور پر متفق |
|  | Did the health worker or peer explore your expectations with this programme?  کیا ہیلتھ ورکر یا رضا کار نے اِس پروگرام سے وابستہ آپکی توقعات کے بارے میں پوچھا ؟ | 0. Strongly disagree مکمل طور پر غیر متفق  1. Disagree غیر متفق  2. Agree متفق  3. Strongly agree مکمل طور پر متفق |
|  | Did your health worker or peer build your hope that your health will get better by participating in the programme?  کیا ہیلتھ ورکر یا رضا کار نے آپ کو امید دلائی کے اِس پروگرام میں شرکت سے آپ کی صحت میں بہتری آئے گی ؟ | 0. Strongly disagree مکمل طور پر غیر متفق  1. Disagree غیر متفق  2. Agree متفق  3. Strongly agree مکمل طور پر متفق |

**Supplementary figure 1: Histogram exhibiting the distribution of empathy scores**


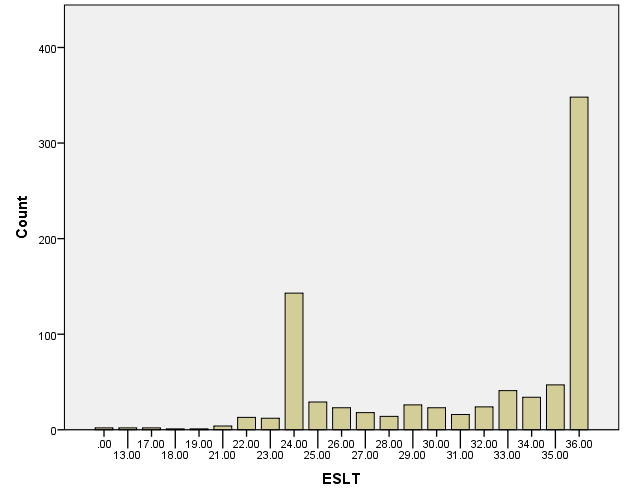


|  | | | |
| --- | --- | --- | --- |
| **Supplementary table 2: Item Statistics** | | | |
|  | Mean | Std. Deviation | N |
| ESLT1 | 2.634 | .5161 | 823 |
| ESLT3 | 2.631 | .5355 | 823 |
| ESLT2 | 2.650 | .5069 | 823 |
| ESLT4 | 2.625 | .5504 | 823 |
| ESLT5 | 2.621 | .5240 | 823 |
| ESLT6 | 2.601 | .5418 | 823 |
| ESLT7 | 2.605 | .5320 | 823 |
| ESLT8 | 2.594 | .5272 | 823 |
| ESLT9 | 2.595 | .5406 | 823 |
| ESLT10 | 2.589 | .5257 | 823 |
| ESLT11 | 2.581 | .5519 | 823 |
| ESLT12 | 2.581 | .5362 | 823 |

| **Supplementary table 3: Inter-Item Correlation Matrix** | | | | | | | | | | | |  |  |
| --- | --- | --- | --- | --- | --- | --- | --- | --- | --- | --- | --- | --- | --- |
|  | ESLT1 | ESLT3 | ESLT2 | ESLT4 | ESLT5 | ESLT6 | ESLT7 | ESLT8 | ESLT9 | ESLT10 | ESLT11 | | ESLT12 |
| ESLT1 | 1.000 | .743 | .854 | .677 | .692 | .640 | .643 | .634 | .598 | .629 | .563 | | .579 |
| ESLT3 | .743 | 1.000 | .792 | .829 | .775 | .716 | .709 | .679 | .635 | .653 | .587 | | .612 |
| ESLT2 | .854 | .792 | 1.000 | .710 | .746 | .652 | .673 | .652 | .641 | .638 | .588 | | .601 |
| ESLT4 | .677 | .829 | .710 | 1.000 | .822 | .754 | .748 | .728 | .675 | .677 | .607 | | .645 |
| ESLT5 | .692 | .775 | .746 | .822 | 1.000 | .821 | .789 | .772 | .716 | .693 | .623 | | .668 |
| ESLT6 | .640 | .716 | .652 | .754 | .821 | 1.000 | .846 | .826 | .765 | .727 | .661 | | .693 |
| ESLT7 | .643 | .709 | .673 | .748 | .789 | .846 | 1.000 | .877 | .802 | .772 | .666 | | .703 |
| ESLT8 | .634 | .679 | .652 | .728 | .772 | .826 | .877 | 1.000 | .819 | .767 | .656 | | .697 |
| ESLT9 | .598 | .635 | .641 | .675 | .716 | .765 | .802 | .819 | 1.000 | .806 | .703 | | .715 |
| ESLT10 | .629 | .653 | .638 | .677 | .693 | .727 | .772 | .767 | .806 | 1.000 | .756 | | .765 |
| ESLT11 | .563 | .587 | .588 | .607 | .623 | .661 | .666 | .656 | .703 | .756 | 1.000 | | .746 |
| ESLT12 | .579 | .612 | .601 | .645 | .668 | .693 | .703 | .697 | .715 | .765 | .746 | | 1.000 |

| **Supplementary table 4: Item-Total Statistics** | | | | | |
| --- | --- | --- | --- | --- | --- |
|  | Scale Mean if Item Deleted | Scale Variance if Item Deleted | Corrected Item-Total Correlation | Squared Multiple Correlation | Cranach’s Alpha if Item Deleted |
| ESLT1 | 28.673 | 25.588 | .764 | . | .965 |
| ESLT2 | 28.657 | 25.500 | .798 | . | .964 |
| ESLT3 | 28.677 | 25.156 | .819 | . | .964 |
| ESLT4 | 28.683 | 24.944 | .836 | . | .963 |
| ESLT5 | 28.687 | 25.033 | .865 | . | .963 |
| ESLT6 | 28.706 | 24.882 | .864 | . | .963 |
| ESLT7 | 28.702 | 24.898 | .878 | . | .962 |
| ESLT8 | 28.713 | 25.008 | .864 | . | .963 |
| ESLT9 | 28.712 | 25.023 | .837 | . | .963 |
| ESLT10 | 28.718 | 25.144 | .839 | . | .963 |
| ESLT11 | 28.727 | 25.345 | .755 | . | .966 |
| ESLT12 | 28.727 | 25.316 | .786 | . | .965 |


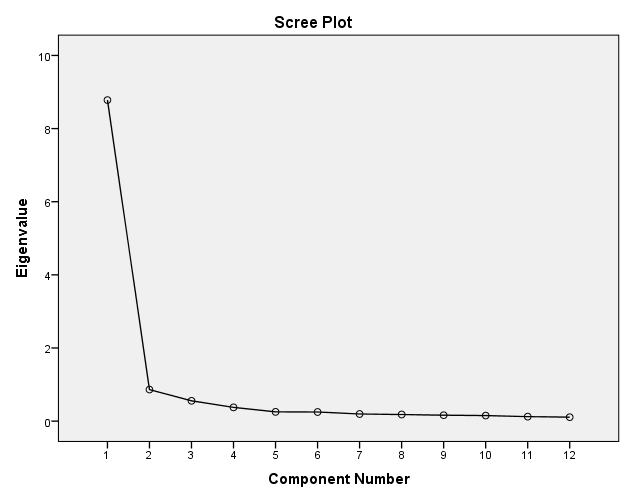


Supplementary figure 2: Scree plot

Supplementary Figure 3: Competency assessment in non-specific therapy ingredients over time among the peers


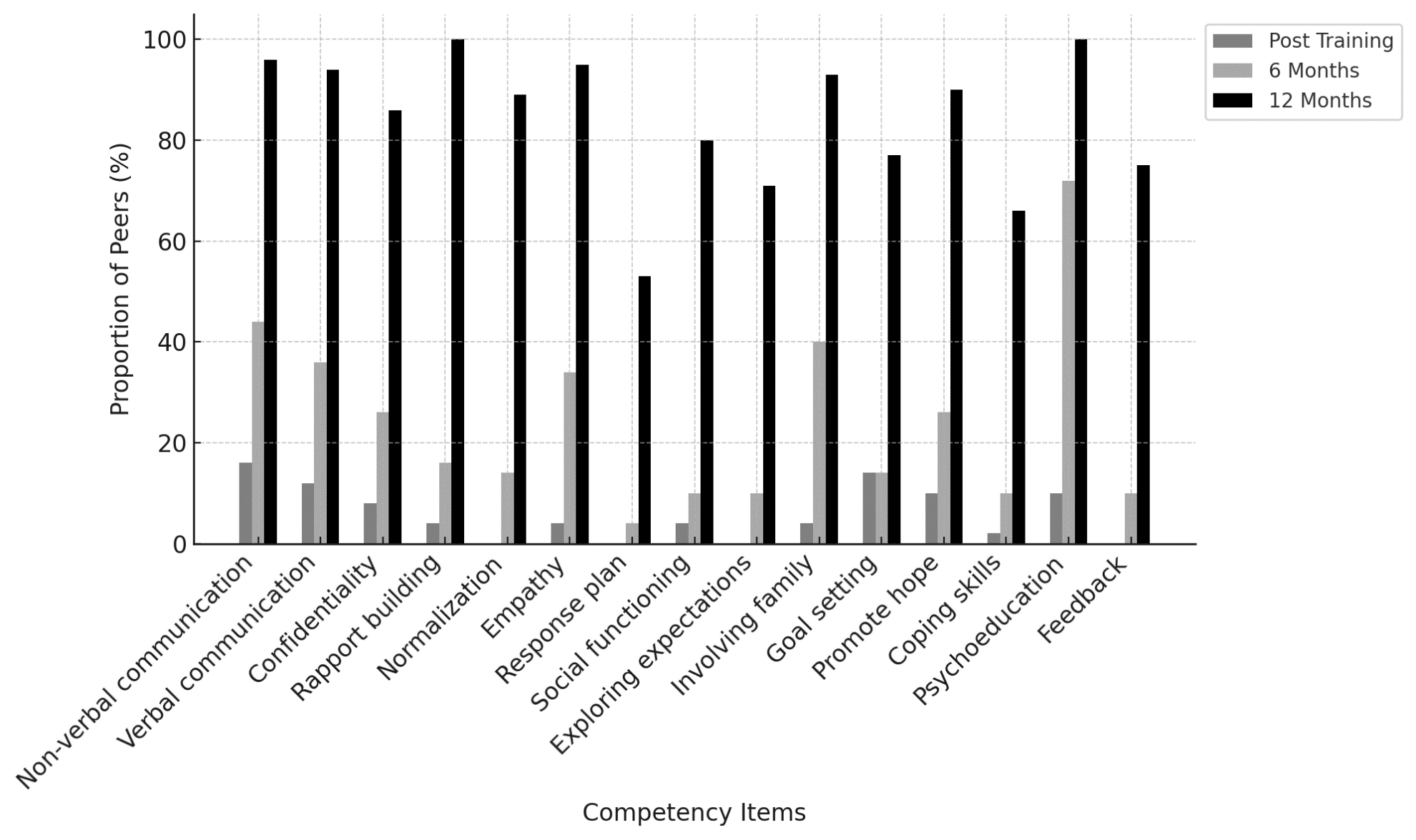


|  |
| --- |
| . |
